# Effect of Green Tea Extract on Systemic Metabolic Homeostasis in Diet-Induced Obese Mice Determined via RNA-Seq Transcriptome Profiles

**DOI:** 10.3390/nu8100640

**Published:** 2016-10-14

**Authors:** Ji-Young Choi, Ye Jin Kim, Ri Ryu, Su-Jung Cho, Eun-Young Kwon, Myung-Sook Choi

**Affiliations:** 1 Department of Food Sciences and Nutrition, Kyungpook National University, 1370 Sankyuk Dong Puk-Ku, Daegu 702-701, Korea; jyjy31@hanmail.net (J.-Y.C.); freewilly59@hanmail.net (Y.J.K.); sangsang0119@gmail.com (R.R.); chocrystalhihi@hanmail.net (S.-J.C.); savage20@naver.com (E.-Y.K.); 2 Center for Food and Nutritional Genomics Research, Kyungpook National University, 1370 Sankyuk Dong Puk-Ku, Daegu 702-701, Korea

**Keywords:** energy expenditure, green tea extract, obesity, RNA-seq, transcriptome

## Abstract

Green tea (GT) has various health effects, including anti-obesity properties. However, the multiple molecular mechanisms of the effects have not been fully determined. The aim of this study was to elucidate the anti-obesity effects of GT via the analysis of its metabolic and transcriptional responses based on RNA-seq profiles. C57BL/6J mice were fed a normal, high-fat (60% energy as fat), or high-fat + 0.25% (*w*/*w*) GT diet for 12 weeks. The GT extract ameliorated obesity, hepatic steatosis, dyslipidemia, and insulin resistance in diet-induced obesity (DIO) mice. GT supplementation resulted in body weight gain reduction than mice fed high-fat through enhanced energy expenditure, and reduced adiposity. The transcriptome profiles of epididymal white adipose tissue (eWAT) suggested that GT augments transcriptional responses to the degradation of branched chain amino acids (BCAAs), as well as AMP-activated protein kinase (AMPK) signaling, which suggests enhanced energy homeostasis. Our findings provide some significant insights into the effects of GT for the prevention of obesity and its comorbidities. We demonstrated that the GT extract contributed to the regulation of systemic metabolic homeostasis via transcriptional responses to not only lipid and glucose metabolism, but also amino acid metabolism via BCAA degradation in the adipose tissue of DIO mice.

## 1. Introduction

Obesity is a metabolic disorder characterized by excess fat accumulation in the body. It is associated with the development of hyperlipidemia, insulin resistance, type 2 diabetes mellitus, hypertension, and non-alcoholic fatty liver disease [1]. A major proportion of the excess body weight in obese subjects is attributable to the expansion of white adipose tissue (WAT). In obesity, WAT is more closely linked to metabolic complications than other tissues [2], while chronic inflammation in the WAT may play a vital role in the development of obesity related metabolic dysfunction [3]. Meanwhile, adipose tissue is considered as a regulator of energy homeostasis and a key endocrine organ secreting multiple adipokines. These adipokines may contribute to the whole-body homeostasis in normal conditions; however, in an obese state, enlarged adipose tissue leads to the dysregulated secretion of adipokines. The dysregulated production of adipokines in obesity may contribute not only to inflammation, but also to the development of various metabolic diseases via altered lipid and glucose homeostasis [4,5]. While the role of adipose tissue in glucose and lipid metabolism is relatively well known, its role in protein and amino acid metabolism is less well recognized. Several studies provide evidence that adipose tissue also contributes to amino acid metabolism, particularly that of branched chain amino acids (BCAAs) [6,7]. Metabolic diseases are characterized by higher levels of circulating BCAAs, which have recently been recognized as regulators of metabolic homeostasis [7,8,9]. Moreover, BCAA supplementation or BCAA deficiency is closely associated with the regulation of metabolic homeostasis [10,11].

Green tea (GT) has been widely studied for its health benefits in humans and animals. It contains caffeine and polyphenolic compounds known as catechins. The four major flavonoids in GT are the catechins such as epicatechin (EC), epigallocatechin (EGC), epicatechin gallate (ECG), and epigallocatechin gallate (EGCG). The efficacy of GT may be attributed to the presence of catechin polyphenols, and it has been suggested that EGCG could be responsible for the various health effects associated with GT [12,13]. GT has been reported to have various effects including anti-obesity, antioxidant, anti-hypertensive, anti-diabetic, and anti-inflammatory [13,14,15,16,17]. In particular, GT has been shown to increase energy expenditure [18] and enhance the metabolic rate and fat-burning ability [19,20]. Most recently, Rocha and colleagues [21] reported that GT extract activates AMP-activated protein kinase (AMPK) and ameliorates WAT metabolic dysfunction that is induced by obesity.

In the present study, we investigated the possible mechanisms of the anti-obesity effect of GT extract by focusing on its phenotypic and transcriptional responses in an obesogenic animal model. This is the first report on the efficacy of GT, with epididymal white adipose tissue (eWAT) and liver tissue transcriptomes obtained from RNA-seq.

## 2. Materials and Methods

### 2.1. Animals

Thirty 4-week-old male C57BL/6J mice were obtained from The Jackson Laboratory (Bar Harbor, ME, USA). All mice were individually housed at a constant temperature (24 °C) and with 12-h light/dark cycles. The mice were fed a normal chow diet for an acclimatization period of 1 week after their arrival. At 5 weeks of age, they were randomly divided into 3 groups of 10 mice per group, and fed either a normal diet (ND), high-fat diet (HFD), or HFD + 0.25% (*w*/*w*) GT extract for 12 weeks. The ND (AIN-93G, TD94045, Harlan, Madison, WI, USA) contained 17.2% kcal from fat, 18.8% kcal from protein, and 63.9% kcal from carbohydrate, while the HFD (TD06414, Harlan, Madison, WI, USA) contained 60.3% kcal from fat, 18.4% kcal from protein, and 21.3% kcal from carbohydrate. GT ethanol extract was obtained from Bioland (Ansan, Korea) and it was a functional food ingredient approved by the Ministry of Food and Drug Safety (MFDS, formerly known as the Korea Food & Drug Administration (KFDA)). The GT extract contained 40.5% catechins, which comprised 4.8% EC, 11.16% EGC, 3.16% ECG, and 21.33% EGCG, and 3.39% caffeine. The human dose of GT, determined based on the MFDS guidelines, is 500 mg/day for adults as catechin. The human GT dose was converted to a mouse dose using the body surface area normalization method [22]. The mice were provided free access to food and distilled water, while food intake, and body weight were measured daily and weekly, respectively. At the end of the diet period, all mice were anesthetized with isoflurane after a 12-h fast. Blood was taken from the inferior vena cava for determination of glucose, plasma lipid, and hormone concentrations. The liver and adipose tissue were removed, rinsed with physiological saline, weighed, immediately frozen in liquid nitrogen, and then stored at −70 °C until use. The animal study protocols were approved by the Ethics Committee of Kyungpook National University (KNU 2012-136).

### 2.2. Measurement of Energy Expenditures

Energy expenditure was measured using an indirect calorimeter (Oxylet; Panlab, Cornella, Spain) in five randomly selected mice per group during final week. The mice were placed into individual metabolic chambers at 25 °C, with free access to food and water. Oxygen and carbon dioxide analyzers were calibrated with high-purity gas. Oxygen consumption and carbon dioxide production were recorded at 3-min intervals, using a computer-assisted data acquisition program, (Chart 5.2; AD Instrument, Sydney, Australia) over a 24-h period, and the data were averaged for each mouse. Energy expenditure (EE) was calculated according to the following formula:

EE (kcal·day^−1^·bodyweight^−0.75^) = VO_2_ × 1.44 × (3.815 + (1.232 × VCO_2_/VO_2_)) (1)

### 2.3. Analysis of Plasma and Hepatic Lipids

Enzymatic assays to determine the plasma free fatty acid, total cholesterol and triglyceride levels were performed using kits purchased from Asan Pharm Co. (Seoul, Korea). Hepatic lipid was extracted according to the methods described by Folch [23]. This was followed by the determination of cholesterol and triglyceride levels using the same enzymatic kit used for the plasma analyses. The hepatic fatty acid level was measured using the Wako enzymatic kit (Wako Chemicals, Richmond, VA, USA). Plasma and hepatic lipids measurements were performed in triplicate.

### 2.4. Levels of Plasma Aspartate Aminotransferase (AST) and Alanine Aminotransferase (ALT)

AST and ALT activities were measured in triplicate using commercially available kits (Asan Pharm Co., Seoul, Korea).

### 2.5. Plasma Glucose and Insulin Resistance Index

The plasma glucose level was measured in triplicate using commercially available kit (Asan Pharm Co., Seoul, Korea). The homeostasis model assessment for insulin resistance (HOMA-IR) was calculated using the following formula:

HOMA-IR = (fasting insulin concentration (mU/L)) × (fasting glucose concentration (mg/dL) × 0.05551)/22.5
(2)

### 2.6. Plasma Hormones, Adipokines, and Proinflammatory Cytokines

Plasma concentrations of hormones (insulin and glucagon) and adipokines (leptin, resistin, and plasminogen activator inhibitor 1 (PAI-1)) were quantified in triplicate using a multiplex detection kit (171-F7001M, Bio-Rad, Hercules, CA, USA) according to the manufacturer’s protocol. Plasma concentrations of adiponectin and plasma cytokines (interferon γ (IFN-γ), monocyte chemoattractant protein 1 (MCP-1), and tumor necrosis factor α (TNF-α)) were quantified in triplicate using a detection kit (171-F7002M, Bio-Rad, Hercules, CA, USA) and multiplex detection kit (M60-009RDPD, Bio-Rad), respectively, according to the manufacturer’s instructions.

### 2.7. Hepatic Enzyme Activities and Glycogen Concentration

Fatty acid β-oxidation and malic enzyme activities were measured in triplicate according to previously described protocols [24,25,26]. Microsomal HMG-CoA reductase (HMGCR) activity was measured in triplicate using [^14^C]-HMG-CoA and [^14^C]-Oleoyl CoA as substrates [27]. The hepatic glycogen concentration was determined in triplicate as previously described [28].

### 2.8. Histological Analysis of eWAT and the Liver

The liver and eWAT were excised from each mouse, fixed in 10% (*v*/*v*) paraformaldehyde in PBS, and embedded in paraffin for staining with hematoxylin and eosin (H & E), and Masson’s trichrome (MT) dye. The stained slices were examined under an optical microscope (Zeiss Axioscope) at 200× magnification [29].

### 2.9. RNA Preparation, Library Preparation, and RNA-Seq

The eWAT and liver were collected from three randomly selected mice from each of the ND, HFD, and GT groups. Total RNA was extracted from the eWAT using TRIzol reagent (Invitrogen Life Technologies, NY, USA) according to the manufacturer’s instructions. After synthesizing cDNA libraries, their quality was evaluated using an Agilent 2100 BioAnalyzer (Agilent, CA, USA). The cDNA libraries were quantified using the KAPA Library Quantification Kit (Kapa Biosystems, Boston, MA, USA). After cluster amplification of the denatured templates, samples in flow cells were sequenced as paired-end polymers (2 × 100 bp) using the Illumina HiSeq2500 (Illumina, San Diego, CA, USA).

### 2.10. Preprocessing of the RNA-Seq Data

Low-quality reads were filtered out according to the following criteria: reads containing >10% of skipped bases (marked as N’s), reads containing >40% of bases whose quality scores were <20, and reads whose average quality score was <20. The filtering process was performed using in-house scripts. The remaining reads were mapped onto the mouse reference genome (Ensembl, release 72), using the aligner software STAR version 2.3.0e [30]. The gene expression levels were measured using Cufflinks version 2.1.1 [31], using the gene annotation database of Ensembl, release 72. The noncoding gene regions were removed by means of the mask option. To improve the accuracy of the measurement, “multiread correction” and “frag bias-correct” options were used. All other options were set to the default values.

### 2.11. Differential Transcriptome and Functional Analysis

For differential expression analysis, the data on gene level counts were generated using HTSeq-count version 0.5.4p3 [32]. Using the resulting read count data, differentially expressed genes (DEGs) were identified using the R software package, TCC (Bioconductor open source project) [33]. The TCC package uses robust normalization strategies to compare tag count data. Normalization factors were calculated using the iterative DEGES/edgeR method. The *q*-value was calculated from the *p* value using the p.adjust function in the R package and the default settings. DEGs were identified based on a *q*-value threshold of less than 0.05. K-means clustering was performed in the Bioinformatics Toolbox of MATLAB R2009a.

### 2.12. Molecular Pathway and Function Analysis

The DEG lists were analyzed using the Ingenuity Pathway Analysis (IPA) software (IPA, Ingenuity^®^ systems, Qiagen, CA, USA). IPA allows for the identification of network interactions and pathway interactions between genes, based on an extensive manually curated database of published gene interactions. We uploaded the genes with a *q*-value threshold of less than 0.05, and a fold change in expression of more than 1.5, after HFD, with or without GT supplementation, and the associated expression value from the RNA-seq data into IPA.

### 2.13. Statistical Analysis

Results were expressed as the mean ± standard error of the mean (SEM). Differences among the ND, HFD, and GT groups were assessed for significance using one-way analysis of variance (one-way ANOVA), as calculated using the SPSS v18.0 software (SPSS Inc., Chicago, IL, USA). Any differences identified between groups at each time-point were analyzed further using Duncan’s multiple-range post-hoc test. Results were considered statistically significant at *p* < 0.05.

## 3. Results

### 3.1. Supplementation with GT Ethanol Extract Reduced Body Weight Gain and Body Fat Mass with Enhanced Energy Expenditure and Plasma Lipid and Glucose Profiles in Diet-Induce Obesity (DIO) Mice

**Figure 1:**
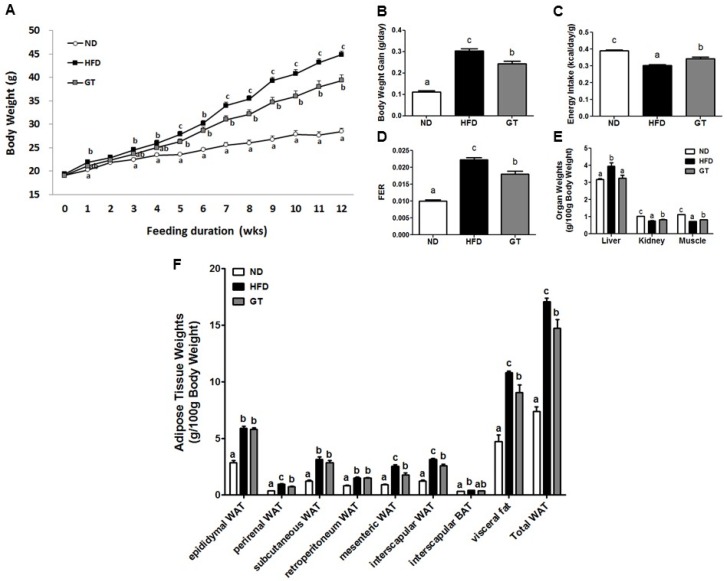
(**A**) Changes in body weight over 12 weeks; (**B**) body weight gain; (**C**) differences in energy intake; (**D**) food efficiency ratio; (**E**) organ weight; and (**F**) adipose tissue (AT) weights in diet-induced obese C57BL/6J mice treated with green tea extract for 12 weeks. The data are shown as mean ± standard error of the mean. ^a–c^ Mean values not sharing a common superscript were significantly different among the groups (*p* < 0.05). ND, normal diet, AIN-93G; HFD, high-fat diet, 60% kcal from fat; GT, green tea extract (0.25%, *w*/*w*). FER, Food efficiency ratio = body weight gain/energy intake per day. Energy intake (kcal/day/g) = food intake (g/day) × calories (kcal/g) × body weight (g). The energy intake was normalized by mouse body weight.

Body weight and body weight gain were significantly lower in the GT group than in the HFD group (Figure 1A,B). For this reason, the food efficiency ratio (FER) was significantly lower in the GT group than in the HFD group (Figure 1D). Similar to the trends observed in body weight, liver weight per 100 g of body weight was significantly lower in the GT-treated group than in the HFD group. The significant reductions in kidney and muscle weights observed in the HFD group were reversed upon treatment with GT (Figure 1E). Furthermore, treatment with GT resulted in significant decreases in the weights of perirenal, mesenteric, interscapular, and visceral tissue, in addition to total WAT when compared with the HFD group (Figure 1F).

**Figure 2:**
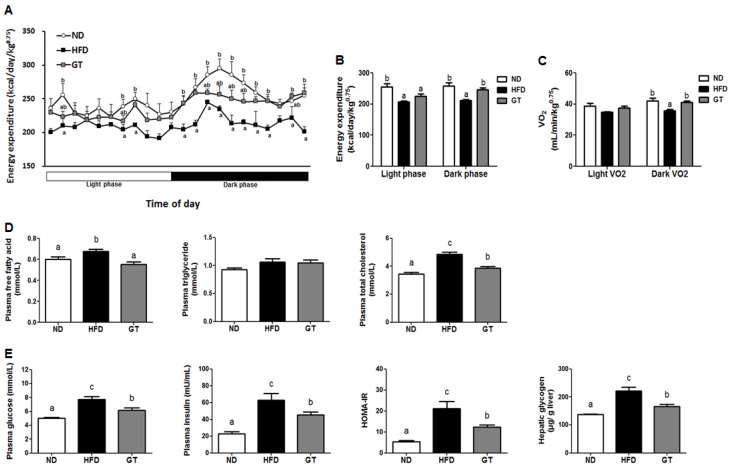
(**A**,**B**) Energy expenditure; (**C**) oxygen consumption (VO_2_); (**D**) plasma lipid profiles; and (**E**) glucose metabolism-related markers in diet-induced obese C57BL/6J mice treated with green tea extract for 12 weeks. The data are shown as mean ± standard error of the mean. ^a–c^ Mean values not sharing a common superscript were significantly different among the groups (*p* < 0.05). ND, normal diet, AIN-93G; HFD, high-fat diet, 60% kcal from fat; GT, green tea extract (0.25%, *w*/*w*).

The energy expenditure decreased in the HFD group relative to the ND group during both light and dark phases, while GT supplementation significantly augmented the energy expenditure during the dark phase (Figure 2A,B). Furthermore, GT-treated mice exhibited higher oxygen consumption (VO_2_) than HFD-fed mice during the dark phase (Figure 2C). Plasma-free fatty acid and total-cholesterol levels were significantly lower in the GT group than in the HFD group (Figure 2D). Plasma glucose and insulin levels were also significantly reduced with GT supplementation after 12 weeks compared to that in the HFD group. Additionally, the HOMA-IR was significantly lower in the GT group than in the HFD group, which indicates decreased insulin resistance. The HFD-induced elevation in hepatic glycogen was attenuated by GT supplementation (Figure 2E).

### 3.2. GT Ethanol Extract Attenuated the Level of Plasma Adipokines in DIO Mice and Modulated Transcriptional Responses to a HFD in eWAT

**Figure 3:**
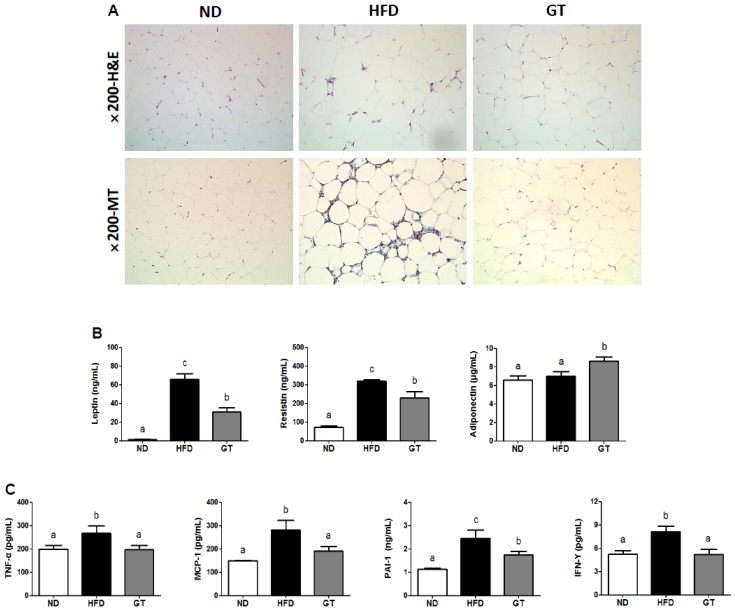
(**A**) Hematoxylin and eosin staining (H & E; upper panel) and Masson’s trichrome staining (MT; lower panel) of epididymal adipocytes (magnification 200×); and (**B**,**C**) differences in plasma adipokines in diet-induced obese C57BL/6J mice treated with green tea extract for 12 weeks. The data are shown as mean ± standard error of the mean. ^a–c^ Mean values not sharing a common superscript were significantly different among the groups (*p* < 0.05). ND, normal diet, AIN-93G; HFD, high-fat diet, 60% kcal from fat; GT, green tea extract, 0.25% *w*/*w*.

The epididymal adipocyte size in the HFD group was visibly larger than in the ND-fed mice. Treatment with GT reduced the epididymal adipocyte size when compared to the size in HFD-fed mice. According to the results of MT staining, HFD-fed mice exhibited visible morphological evidence of fibrosis when compared to the ND-fed mice, while no signs of fibrotic changes were identified in the GT group (Figure 3A). The plasma leptin and resistin levels were remarkably lower in GT-treated mice than in the HFD-fed mice. In contrast, plasma adiponectin levels were significantly elevated in the GT group (Figure 3B) than in the ND and HFD groups. Furthermore, GT supplementation resulted in a significant decrease in the plasma levels of tumor necrosis factor α (TNF-α), monocyte chemoattractant protein 1 (MCP-1), plasminogen activator inhibitor 1 (PAI-1), and interferon γ (IFN-γ) when compared to HFD group (Figure 3C).

**Figure 4:**
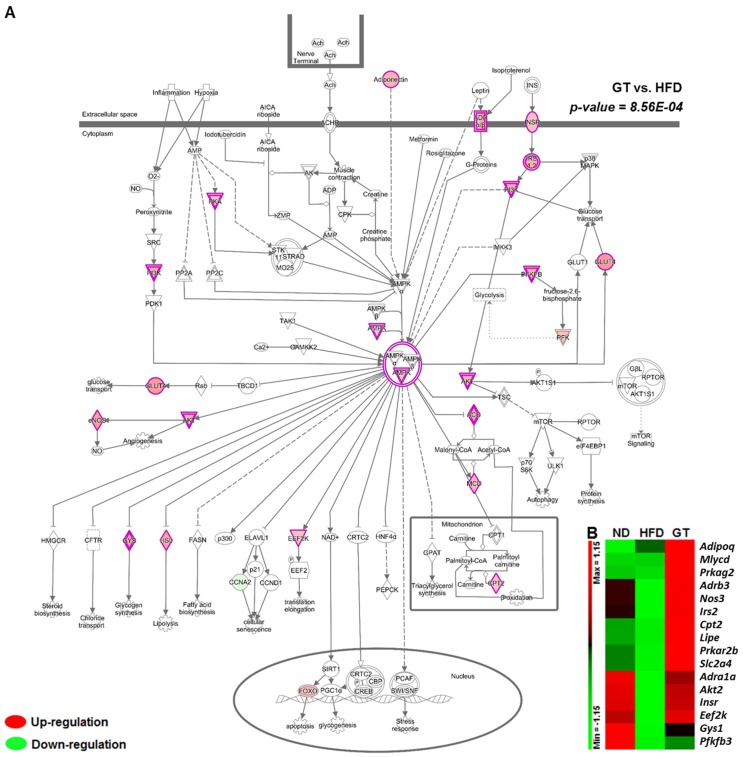
(**A**) Canonical pathway; and (**B**) heat map of the genes related to the AMP-activated protein kinase (AMPK) signaling pathway in epididymal white adipose tissue (eWAT) of diet-induced obese C57BL/6J mice treated with green tea extract for 12 weeks. ND, normal diet, AIN-93G; HFD, high-fat diet, 60% kcal from fat; GT, green tea extract, 0.25% *w*/*w*. The significant pathways and functions were obtained via Ingenuity Pathway Analysis (IPA).

**Figure 5:**
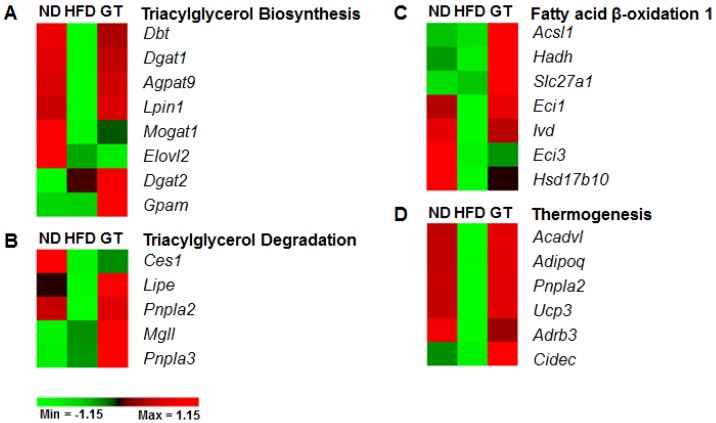
Expression profiles of lipid metabolism-related genes in epididymal white adipose tissue (eWAT) of diet-induced obese C57BL/6J mice treated with green tea extract for 12 weeks. ND, normal diet, AIN-93G; HFD, high-fat diet, 60% kcal from fat; GT, green tea extract, 0.25% *w*/*w*. The significant pathways and functions were obtained via Ingenuity Pathway Analysis (IPA).

To identify the global transcriptomic profiles associated with obesity and its comorbidities, we performed RNA-seq on eWAT and liver samples obtained from the ND, HFD, and GT groups and systematically analyzed the results. First, we identified differentially expressed genes (DEGs) between HFD-fed and GT-treated mice using the cutoff set to a fold change of ≥1.5 and a *q*-value of <0.05. In the eWAT, 1173 DEGs were identified between GT-treated and HFD-fed mice (703 upregulated and 470 downregulated). Next, we identified significant molecular pathways and functions when comparing GT to HFD groups using ingenuity pathway analysis (IPA). GT supplementation resulted in the up-regulation of AMPK signaling-related genes such as *Acacb*, *Adipoq*, *Adra1a*, *Adrb3*, *Akt2*, *Cpt2*, *Eef2k*, *Gys1*, *Insr*, *Irs2*, *Lipe*, *Mlycd*, *Nos3*, *Pfkfb3*, *Prkag2*, *Prkar2b*, and *Slc2a4* of adipose tissue in DIO mice (Figure 4A,B). Among the significant canonical pathways, triacylglycerol biosynthesis and degradation, and fatty acid β-oxidation pathway related genes, were up-regulated by GT supplementation (Figure 5A–C). Furthermore, GT supplementation increased the transcriptional response involved in thermogenesis (Figure 5D).

**Figure 6:**
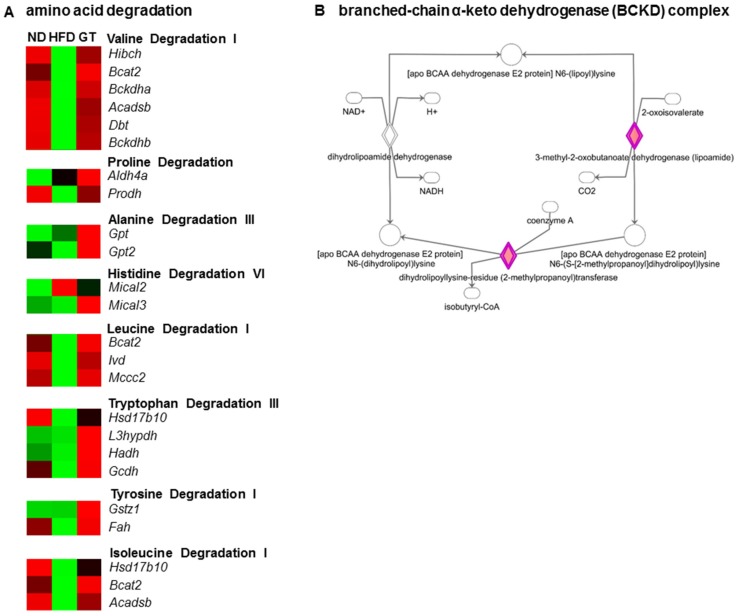
(**A**) A heat map of the genes involved in amino acid degradation; and (**B**) the transcriptional response related to branched-chain α-keto dehydrogenase (BCKD) in the epididymal white adipose tissue (eWAT) of diet-induced obese C57BL/6J mice treated with green tea extract for 12 weeks. The significant pathways were obtained via Ingenuity Pathway Analysis (IPA).

Degradation pathways of amino acids, including valine, proline, alanine, histidine, leucine, tryptophan, tyrosine, and isoleucine, were also significantly altered by GT supplementation. It augmented the transcriptional response to the degradation of BCAA in the eWAT of DIO mice, and valine degradation was identified as the most significant canonical pathway among 298 canonical pathways based on IPA. The expression of genes related to the degradation of leucine and isoleucine was also up-regulated by GT supplementation (Figure 6A). In particular, mRNA expression of the branched-chain α-keto dehydrogenase (BCKD) complex components (*Bckdha*, *Bckdhb*, and *Dbt*) of the eWAT in DIO mice was significantly up-regulated by GT supplementation (Figure 6B).

### 3.3. GT Extract Ameliorated Hepatic Steatosis via the Metabolic and Transcriptional Responses in the Livers of DIO Mice

**Figure 7:**
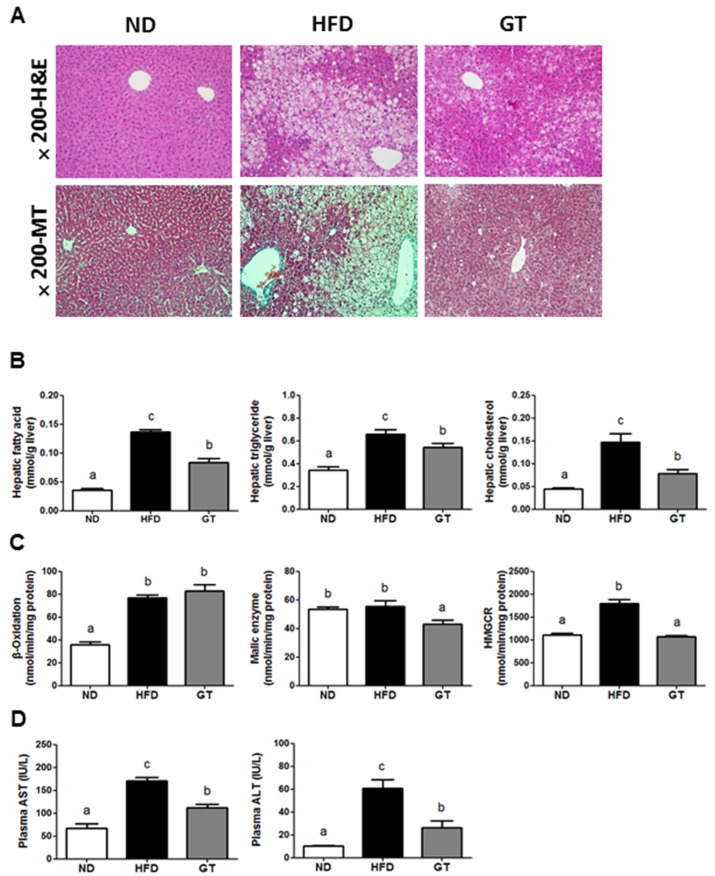

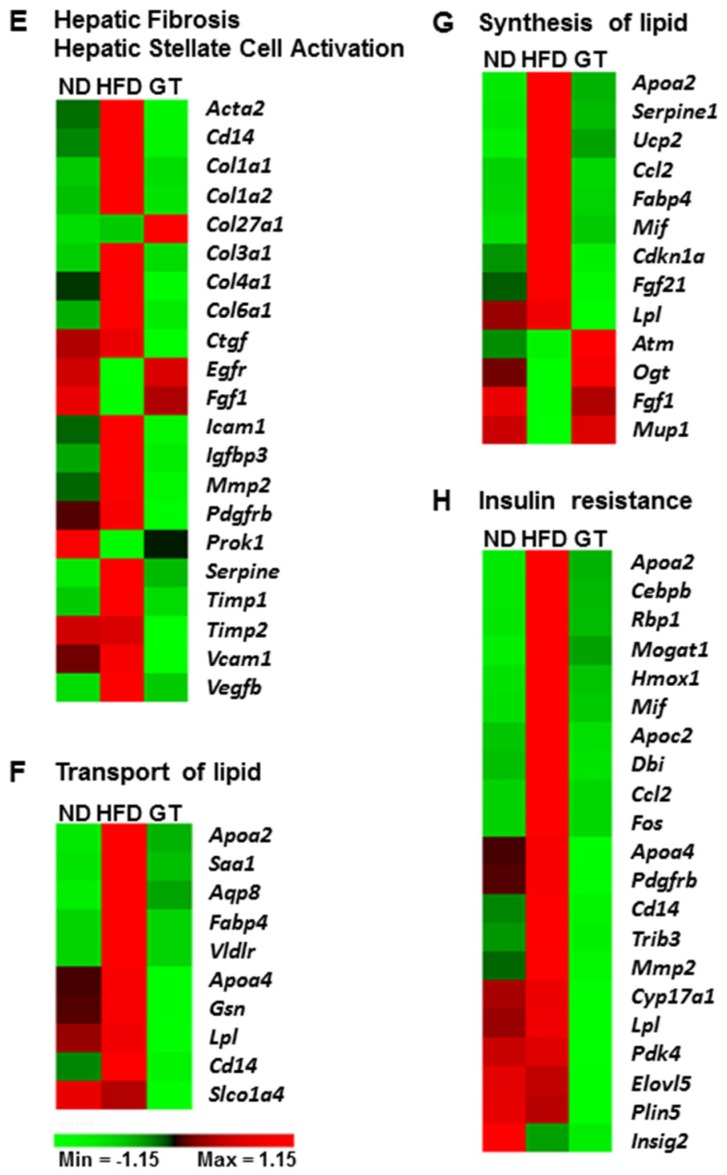
(**A**) Hematoxylin and eosin staining (H & E, upper panel) and Masson’s trichrome (MT, lower panel) staining of the liver (magnification 200×); (**B**) hepatic lipid profiles; (**C**) hepatic activities of lipid-regulating enzymes; (**D**) plasma aspartate aminotransferase (AST) and alanine aminotransferase (ALT) levels; and (**E**–**H**) Heat map of the genes involved in hepatic fibrosis/hepatic stellate cell activation, transport of lipid, synthesis of lipid and insulin resistance in the liver of diet-induced obese C57BL/6J mice treated with green tea extract for 12 weeks. The data are shown as mean ± standard error of the mean. ^a–c^ Mean values not sharing a common superscript were significantly different among the groups (*p* < 0.05). ND, normal diet, AIN-93G; HFD, high-fat diet, 60% kcal from fat; GT, green tea extract, 0.25% *w*/*w*. The significant pathways and functions were obtained via Ingenuity Pathway Analysis (IPA).

The morphology of hepatic tissue revealed a decrease in the accumulation of hepatic lipid droplets in the GT group. MT staining of the liver also demonstrated no fibrotic changes in the ND and GT groups, whereas fibrosis was observed around the vessels in the HFD group (Figure 7A). Hepatic fatty acids, triglyceride, and cholesterol contents of the GT group were significantly lower when compared to the HFD group (Figure 7B). Although there was no significant difference in the activity of β-oxidation between GT and HFD groups, the activities of HMG-CoA reductase (HMGCR) and malic enzyme were markedly reduced in the GT group relative to the HFD group (Figure 7C). Furthermore, the levels of plasma aspartate aminotransferase (AST) and alanine aminotransferase (ALT), markers of hepatic toxicity, were significantly decreased by GT supplementation (Figure 7D).

The RNA-seq data revealed several adaptive molecular functions and pathways that partly explain why GT-treated mice were protected from the pathological conditions present in DIO mice. In the liver, 1561 DEGs were identified between the GT-treated and HFD-fed mice (1119 up-regulated and 442 down-regulated). The most significant identified pathway was the hepatic fibrosis/hepatic stellate cell activation of 289 canonical pathways obtained from IPA. The majority of hepatic fibrosis/hepatic stellate cell activation-associated genes were down-regulated in the liver upon GT-treatment (Figure 7E). Furthermore, GT attenuated transcriptional regulation such as the transport and synthesis of lipids in DIO mice (Figure 7F,G). It also reversed the transcriptional response associated with insulin resistance in the HFD (Figure 7H).

## 4. Discussion

In this study, we have shown the multiple effects of GT extract that are involved in ameliorating metabolic disturbances in DIO mice. GT treatment attenuated HFD-induced obesity, dyslipidemia, hepatic steatosis, insulin resistance, and the inflammatory response. In the current study, we evaluated the effect of GT and the potential mechanisms underlying its metabolic regulation using RNA-seq transcriptomic profiles in a DIO model. The transcriptomic profiles based on RNA-seq revealed several adaptive mechanisms that may explain why GT-treated mice were protected from the pathological changes that occurred in HFD-fed mice.

We were able to find significant canonical pathways and molecular functions via IPA based on the DEGs between GT-treated and HFD-fed mice. In this study, GT supplementation resulted in the up-regulation of AMPK signaling-related genes in the eWAT of DIO mice. In particular, the expressions of *Adipoq* and *Adrb3*, considered to be AMPK activation factors, were significantly augmented and the expression of *Glut4* (also known as *Slc2a4*), a major glucose transporter, was also up-regulated because of GT supplementation. AMPK is a key regulator of cellular energy metabolism and the whole-body energy balance [34]. Previous studies reveal that EGCG, major catechin of green tea extract, and green tea extract activate AMPK in various cell lines and tissues. Several reports suggest that EGCG inhibits adipogenesis through activation of AMPK in 3T3-L1 cells [35,36] and EGCG anti-diabetic effects essentially depended on the AMPK activation in rat L6 muscle cells [37]. Moreover, EGCG prevents fatty liver by AMPK activation via liver kinase B1 inhibiting mediators responsible for the synthesis of fatty acid and de novo lipogenesis in mice fed a HFD [38]. In addition, similar effects of GT supplementation in the prevention of the deleterious effects of HFD have been supported by Rocha et al., whose results suggested that GT extract could improve WAT metabolic dysfunction induced via activation of AMPK [21]. Thus, GT supplementation contributed to the energy homeostasis of adipose tissue via the regulation of the AMPK signaling pathway in DIO mice. Furthermore, GT supplementation promoted the expression of genes involved in lipolysis, fatty acid oxidation, and thermogenesis in adipose tissue. Despite upregulating transcriptional pathways involved in triacylglycerol biosynthesis in adipose tissue, GT markedly reduced the WAT weights. It is plausible that GT restricts triglyceride availability by increasing lipolysis, oxidation, and thermogenesis prior to lipid droplet formation in adipose tissue. These transcriptional responses in the WAT suggest that although GT activates lipogenesis, it also simultaneously increases lipolysis, fatty acid oxidation, and thermogenesis, which may contribute to the reduction in adiposity.

Another possible explanation for the observed body fat reduction could be the increased transcriptional response of BCAA degradation upon treatment with GT when compared to the HFD group. Based on the IPA, the transcriptional profiles of the eWAT of GT-treated DIO mice were strongly linked with amino acid degradation, particularly BCAAs. The BCAAs, leucine, isoleucine, and valine, are three of the nine essential amino acids that have been recently recognized as regulators of metabolic homeostasis [8]. Several studies have reported that abnormal BCAA levels are associated with various metabolic diseases in both humans and rodents [6,9,39]. BCAA homeostasis is mainly controlled by BCKD, the rate-limiting enzyme in BCAA catabolism [40], the expression of which is reduced in an obese state [6]. While the role of adipose tissue in glucose and lipid metabolism is relatively well known, its role in protein and amino acid metabolism is less well recognized. Several studies have provided evidence of adipose tissue contributing to amino acid metabolism, particularly BCAAs [6,7]. In this study, GT supplementation augmented the mRNA expression of the BCKD complex components, *Bckdha*, *Bckdhb*, and *Dbt*, in the eWAT of DIO mice. A previous study reported that WAT BCKD protein was significantly reduced in various obesity models (fa/fa rats, *db*/*db* mice, and DIO mice), and that BCKD component transcripts were significantly lower in adipocytes from obese versus lean subjects [6]. Lian and colleagues [41] reported that impaired adiponectin signaling contributed to the disturbed catabolism of BCAA in diabetic mice. In the current study, both mRNA expression of *Adipoq* in the eWAT, and plasma adiponectin levels, were markedly up-regulated by GT supplementation in DIO mice. Therefore, it is thought that the GT extract contributes to whole-body homeostasis partly via increased transcriptional response to the degradation of BCAAs in the eWAT of DIO mice.

GT supplementation increased energy expenditure during the dark phase, without a difference in the energy intake of DIO mice. Accordingly, GT-treated mice were more metabolically active than HFD-fed mice, which was again reflected in their lower body weight and body fat mass. Consistent with the reduced adiposity, GT supplementation improved endocrine secretion, including a reduction in chemokines, cytokines, and hormone levels in mice fed an HFD. Plasma leptin and resistin levels were higher in HFD-fed mice than in ND-fed mice; however, this change was attenuated in GT-treated mice. In contrast, plasma adiponectin, a regulator of energy homeostasis, was significantly elevated by GT supplementation, with a concomitant increase in the mRNA expression of *Adipoq* in the eWAT. The accumulation of excess body fats was related to the augmentation of inflammatory markers including TNF-α, MCP-1, PAI-1, and IFN-γ. The down-regulation of these markers in GT-treated mice suggests that GT may suppress inflammation and improve immune responses.

HFD commonly induces metabolic alterations, which include dyslipidemia and hepatic steatosis. However, GT supplementation attenuated the plasma and hepatic lipid contents, with decreased hepatic lipogenic enzyme activities (malic enzyme and HMCGR). This suggests that GT may limit hepatic lipid availability by inhibiting lipogenesis, thereby, reducing hepatic lipotoxicity markers such as AST and ALT. A histological examination of liver tissue from GT-treated DIO mice revealed a reduction in lipid droplets when compared with the HFD group, indicating an amelioration of hepatic steatosis. In addition, notable hepatic fibrosis was observed around the vessels in the livers of HFD-fed mice, whereas the livers of mice in the GT group revealed no fibrotic changes. The alteration of these phenotype markers in GT-treated mice was underpinned by the transcriptomic profiles of the liver. Our present data obtained by IPA demonstrate that hepatic fibrosis/hepatic stellate cell activation is the most significant canonical pathway among the 289 canonical pathways. The hepatic stellate cell is the key cellular element involved in the development of hepatic fibrosis [42]. GT supplementation attenuated the expression of hepatic fibrosis/hepatic stellate cell activation-related genes, which was accompanied with the down-regulation of genes for lipid transport and synthesis. Accordingly, these transcriptional responses may contribute to the attenuation of hepatic steatosis as well as fibrosis.

There are limitations to this study. First, we only reported phenotype characteristics and transcriptomic profiles without direct evidence of each potential transcriptional pathway. In addition, it is difficult to distinguish the cause of increased energy expenditure that is product of increased movement, increased resting energy expenditure, or a combination, due to absence of physical activity data. Despite the aforementioned limitations, the present findings provide important insights into the mechanism by which the GT extract exerts its anti-obesity effects and ameliorates metabolic complications such as adiposity, dyslipidemia, hepatic steatosis, and insulin resistance. This modulation occurs partly through an increase in energy expenditure, and via metabolic and transcriptional regulation in the WAT and livers of DIO mice.

## 5. Conclusions

In conclusion, the overall metabolic and transcriptional responses to the GT extract in DIO proved to be desirable. GT contributes to systemic metabolic homeostasis via the transcriptional regulation of BCAA degradation, as well as lipid and glucose metabolism in adipose tissue.

## Supplementary Materials

The following are available online at http://www.mdpi.com/2072-6643/8/10/640/s1, Table S1: Primer sequences used for RT-qPCR, Figure S1: Validation of RNA-seq data by RT-qPCR.

## Abbreviations

ALT	alanine aminotransferase
AMPK	AMP-activated protein kinase
AST	aspartate aminotransferase
BCAAs	branched chain amino acids
BCKD	branched-chain α-keto dehydrogenase
DEGs	differentially expressed genes
DIO	diet-induced obesity
EC	epicatechin
ECG	epicatechin gallate
EGC	epigallocatechin
EGCG	epigallocatechin gallate
eWAT	epididymal white adipose tissue
FER	food efficiency ratio
GT	green tea
HFD	high-fat diet
HMGCR	HMG-CoA reductase
HOMA-IR	homeostasis model assessment for insulin resistance
IFN-γ	interferon γ
IPA	ingenuity pathway analysis
MCP-1	monocyte chemoattractant protein 1
ND	normal diet
PAI-1	plasminogen activator inhibitor 1
TNF-α	tumor necrosis factor α
