# Supplementary material for: Effect of Green Tea Extract on Systemic Metabolic Homeostasis in Diet-Induced Obese Mice Determined via RNA-Seq Transcriptome Profiles
Source: Nutrients. 2016 Oct 14;8(10):640. doi: 10.3390/nu8100640 (PMC5084027; doi:10.3390/nu8100640)
Supplement: Supplementary file 1 [file nutrients-08-00640-s001.docx]

Supplementary Materials: Effect of Green Tea Extract on Systemic Metabolic Homeostasis in Diet-Induced Obese Mice Determined via RNA-Seq
Transcriptome Profiles

Ji-Young Choi, Ye Jin Kim, Ri Ryu, Su-Jung Cho, Eun-Young Kwon and Myung-Sook Choi

RT-qPCR

Total RNA (1 μg) was reverse-transcribed into cDNA using the QuantiTect^®^ reverse transcription kit (Qiagen, Hilden, Germany). The mRNA expression was then quantified by real-time quantitative reverse transcriptase polymerase chain reaction (RT-qPCR), using the SYBR green PCR kit (Qiagen, Hilden, Germany) and the CFX96TM real-time system (Bio-Rad, Foster City, CA, USA). Gene-specific mouse primers were used as presented in Table S1. Cycle threshold (*C*_t_) data was normalized using glyceraldehyde-3-phosphate dehydrogenase (GAPDH), and the relative gene expression was calculated using the 2^−ΔΔ^*^C^*^t^ method [1].

**Table S1.** Primer sequences used for RT-qPCR.

| **Primer** | **Sequence** |
| --- | --- |
| *Adipoq* | 5′-GGTCTTCTTGGTCCTAAGGGTGAG-3′ (forward)  5′-GCGGCTTCTCCAGGCTCTC-3′ (reverse) |
| *Adrb3* | 5′-CTTCACTCTCTGCTGGTTGC-3′ (forward)  5′-AAGGCAGAATTGGCATAACC-3′ (reverse) |
| *Irs2* | 5′-CACAAGTACCTGATCGCCCTCTAC-3′ (forword)  5′-CTCCTGCTCCTGCTCGTTCTC-3′ (reverse) |
| *Lpl* | 5′-GACTCGCTCTCAGATGCCCTAC-3′ (forword)  5′-GCCTGGTTGTGTTGCTTGCC-3′ (reverse) |
| *Slc2a4* | 5′-CCACAGAAGGTGATTGAACAGAGC-3′ (forword)  5′-AGAGCCCAGAGCGTAGTGAGG-3′ (reverse) |
| *Gapdh* | 5′-ACAATGAATACGGCTACAGCAACAG-3′ (forward)  5′-GGTGGTCCAGGGTTTCTTACTCC-3′ (reverse) |

*Adipoq*, adiponectin; *Adrb3*, adrenergic receptor, beta 3; *Irs2*, insulin receptor substrate 2; *Lpl*, lipoprotein lipase; *Slc2a4*, solute carrier family 2 member 4; *Gapdh*, Glyceraldehyde 3-phosphate dehydrogenase.

Epididymal WAT


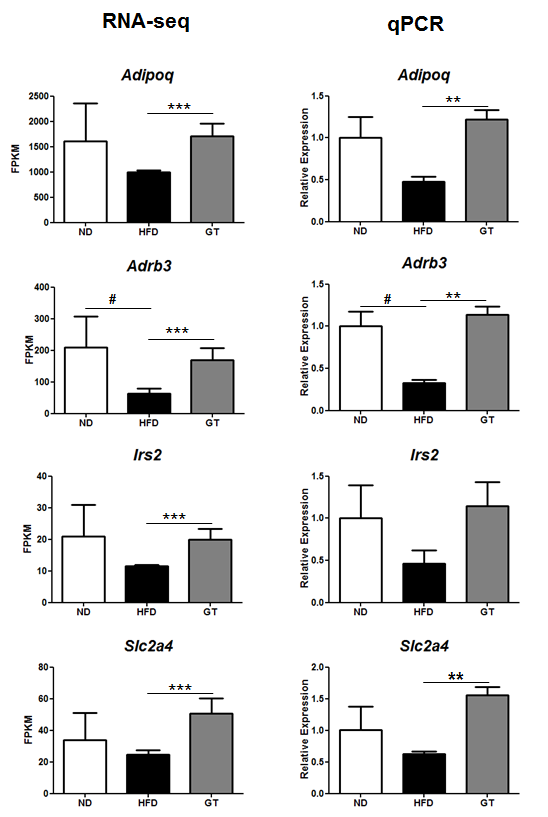


**Figure S1.** *Cont.*

Liver


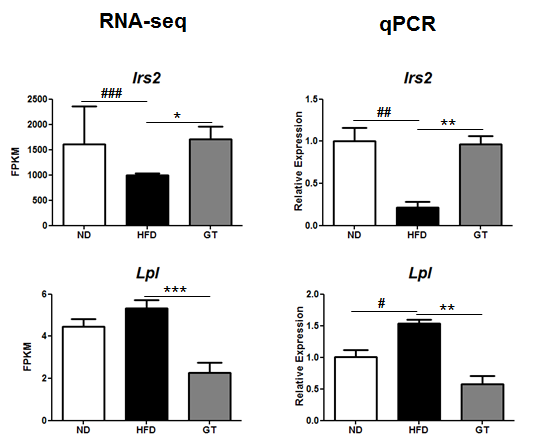


**Figure S1.** Validation of RNA-seq data by RT-qPCR. ND-fed vs. HFD mice: ^#^ *p* < 0.05, ^##^ *p* < 0.01, and ^###^ *p* < 0.001. HFD vs. GT mice: * *p* < 0.05, ** *p* < 0.01, and *** *p* < 0.001.

References

1. Schmittgen, T.D.; Livak, K.J. Analyzing real-time PCR data by the comparative CT method. *Nat. Protoc.*
   **2008**, *3*, 110–1108.
